# Assessing Habitat Suitability and Landscape Connectivity for the Endangered Forest Musk Deer (*Moschus berezovskii*) at Its Northern Distribution Margin: A Multi‐Scale Ensemble Approach

**DOI:** 10.1002/ece3.74086

**Published:** 2026-08-12

**Authors:** Wangshan Zheng, Pengfei Luo, Yixin Li, Luyao Hai, Xuelin Jin, Xinghu Qin, Liping Yan, Defu Hu

**Affiliations:** ^1^ School of Ecology and Nature Conservation Beijing Forestry University Beijing China; ^2^ Shaanxi Institute of Zoology Xi'an China

**Keywords:** conservation gaps, dispersal bottlenecks, forest musk deer, peripheral population, scale‐dependent, thermal refugia

## Abstract

For forest‐dwelling habitat specialists, landscapes at range margins can serve as critical refugia if they provide suitable topographic and thermal conditions. However, the spatial isolation of these important habitats makes their dispersal pathways vulnerable to anthropogenic barriers. Understanding the habitat suitability and landscape connectivity of such peripheral populations is critical for targeted conservation. This study investigates the endangered forest musk deer ( *Moschus berezovskii* ) at its northernmost known distribution limit in China (Huanglong Mountains, Shaanxi). To facilitate representative sampling, we conducted systematic field surveys and utilized DNA molecular identification of fecal remains to collect occurrence data. We employed a multi‐scale ensemble species distribution model (ensemble SDM) and circuit theory to evaluate habitat suitability and landscape connectivity. The results indicate that habitat suitability at this environmental edge is associated with scale‐dependent factors, particularly steep terrain (slope contribution 56.2%), annual precipitation (19.1%), and maximum temperature of the warmest month (12.8%). Our multi‐scale approach reveals a decoupled spatial pattern: forest musk deer rely on macro‐scale steep terrain for security while utilizing micro‐scale microclimatic refugia to mitigate physiological heat stress. We identified 863.0 km^2^ of primary habitat, of which 66.1% is currently protected, revealing substantial conservation gaps. Furthermore, 25 potential dispersal corridors were delineated, showing that localized linear infrastructure intersects with or flanks critical dispersal bottlenecks, impeding landscape permeability. These findings emphasize that conservation planning should shift toward dynamic, network‐oriented management. Future strategies should prioritize expanding protected areas to formally encompass unprotected habitats, mitigating infrastructure barriers via targeted crossing structures, and establishing cross‐administrative governance alongside local community engagement to sustain the long‐term viability of this endangered specialist at its range margins.

## Graphical abstract

We investigated the habitat suitability and landscape connectivity of the endangered forest musk deer ( *Moschus berezovskii* ) at its northernmost known distribution limit using multi‐scale ensemble modeling and circuit theory. Our results highlight a scale‐dependent reliance on macro‐scale steep terrain and micro‐scale thermal refugia, while revealing significant conservation gaps where critical dispersal bottlenecks are impeded by linear infrastructure. These findings emphasize the urgent need to transition toward dynamic, network‐oriented landscape management to ensure the long‐term viability of peripheral populations.

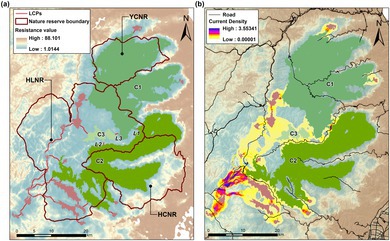

## 1 Introduction

Populations persisting at the periphery of their geographic range (often referred to as edge or peripheral populations) traditionally face substantial physiological and ecological constraints (Lesica and Allendorf 1995; Pironon et al. 2017). However, when specific landscape configurations offer favorable topographic and climatic conditions, these environmental margins can paradoxically serve as critical refugia. To persist in these peripheral areas, species often depend on landscape‐scale features that provide suitable environmental conditions (Keppel et al. 2012; Suggitt et al. 2018). For vagile wildlife, the inherent spatial isolation of these primary habitat patches often severely limits individual movement. Maintaining connectivity between such patches is therefore fundamental to facilitating gene flow, allowing range shifts, and supporting long‐term population viability (Crooks et al. 2017; Keeley et al. 2018). Consequently, elucidating how peripheral populations utilize localized environmental conditions to persist and how their dispersal networks respond to emerging infrastructural barriers has become an important focus for contemporary biodiversity conservation (Rehm et al. 2015; Tucker et al. 2018).

Forest‐dwelling ungulates often exhibit high sensitivity to environmental changes, making them valuable indicator taxa for assessing forest ecosystem connectivity (Feng et al. 2024; Jorge et al. 2020). Predominantly endemic to the montane forests of China, the forest musk deer ( *Moschus berezovskii* ) is a small, solitary ruminant possessing distinctive ecological and morphological traits (Lan et al. 2025; Sheng and Liu 2007). Its coarse, hollow pelage of forest musk deer provides thermal insulation that promotes a preference for cold environments alongside a sensitivity to heat, while an arched body and powerful hind limbs enable navigation across steep terrains. However, how these specialized physiological and morphological traits, specifically thermal intolerance and locomotor agility, interact with local environmental constraints to shape the species' spatial distribution patterns at range margins remains less understood. Historically driven to the brink of extinction by overexploitation for musk and habitat destruction, the forest musk deer is currently listed as Endangered (EN) on the IUCN Red List of Threatened Species (Wang and Harris 2015) and has been designated as a Class I state‐protected species in China since 2002.

Despite numerous conservation planning efforts targeting forest ecosystems globally, existing protected area networks often prioritize overall ecosystem representation or charismatic flagship species (Maxwell et al. 2020; Watson et al. 2014). The specific habitat requirements and landscape connectivity of sensitive species, such as the forest musk deer, are frequently overlooked, particularly at range margins which are often underrepresented in conservation planning, resulting in substantial conservation gaps (Pironon et al. 2017). Furthermore, even in relatively intact mountainous landscapes, linear infrastructure (e.g., road networks), which typically occupies valleys and gentle slopes, frequently conflicts with the dispersal routes of forest‐dwelling species attempting to disperse between primary habitat patches (Ito et al. 2013; Sawyer et al. 2016; Zeller, Wattles, et al. 2020).

The Huanglong Mountains are the northernmost known geographic limit of forest musk deer (Lan et al. 2025; Wu et al. 2011). Although absolute density estimates are currently lacking, converging evidence from our systematic field surveys and reserve monitoring records suggests that this region supports a robust population with relatively high local abundance. When contrasted with the habitat conditions evaluated in several other known ranges (e.g., Gao et al. 2023; Li et al. 2026; Liu et al. 2024), this peripheral stronghold serves as a valuable natural laboratory to investigate the spatial adaptation patterns of the forest musk deer at its range margins. To facilitate a comprehensive understanding of species‐environment relationships rather than solely predicting potential habitats, this study couples a multi‐scale ensemble algorithm with circuit theory to integrate the spatial occurrence of forest musk deer and landscape connectivity into a unified analytical framework. Grounded in the distinctive ecological and morphological traits of this endangered specialist, we aim to address how multi‐scale environmental drivers shape peripheral population of forest musk deer, and how localized anthropogenic barriers impact the species' dispersal network. Therefore, the specific objectives of this study are to (1) identify the environmental drivers defining the habitat suitability of this peripheral stronghold, (2) map primary habitats and quantitatively evaluate the conservation gaps within the existing protected area network, and (3) assess how linear anthropogenic barriers disrupt landscape connectivity and identify critical dispersal bottlenecks. Our findings are expected to provide nuanced ecological insights into the spatial patterns of peripheral forest musk deer populations and offer scientific support for optimizing systematic conservation planning at range margins.

## 2 Materials and Methods

### 2.1 Study Area

**FIGURE 1:**
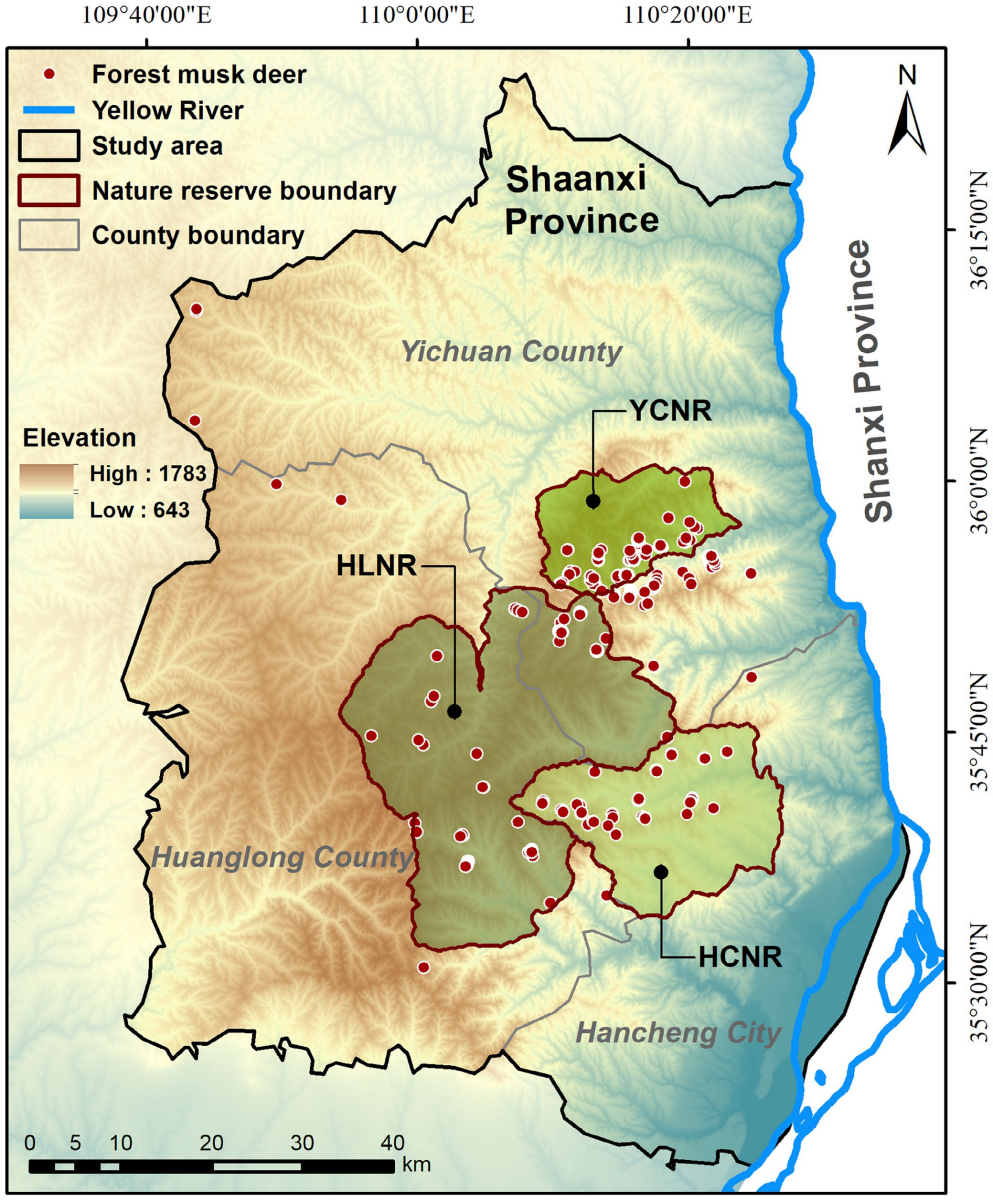
Location of the study area in the Huanglong Mountains, Shaanxi Province. HLNR: Shaanxi Yan'an Huanglong Mountains Brown Eared Pheasant National Nature Reserve; HCNR: Shaanxi Hancheng Huanglong Mountains Brown Eared Pheasant National Nature Reserve; YCNR: Shaanxi Yichuan Siberian Musk Deer Provincial Nature Reserve.

The Huanglong Mountains are located on the southeastern edge of Yan'an City, Shaanxi Province, China (109°38′51.48″–110°36′30″ E, 35°18′47″–36°24′8″ N), covering 7292.8 km^2^ and including three protected areas: Shaanxi Yan'an Huanglong Mountains Brown Eared Pheasant National Nature Reserve (hereinafter HLNR), Shaanxi Hancheng Huanglong Mountains Brown Eared Pheasant National Nature Reserve (HCNR), and Shaanxi Yichuan Siberian Musk Deer Provincial Nature Reserve (YCNR) (Figure 1). This region has a temperate continental monsoon climate, with an annual mean temperature of 8°C–10°C, annual precipitation of 400–600 mm, and elevation ranging from 800 to 1500 m. Vegetation types follow an elevational gradient (broadleaf forest, coniferous‐broadleaf mixed forest, coniferous forest, montane meadow), with a forest coverage rate ≥ 65%. The area supports abundant protected flora and fauna. Dominant sympatric mammals include the leopard ( *Panthera pardus* ), Siberian roe deer ( *Capreolus pygargus* ), and wild boar ( *Sus scrofa* ); dominant sympatric birds include the brown eared pheasant ( *Crossoptilon mantchuricum* ), golden pheasant ( *Chrysolophus pictus* ), and golden eagle ( *Aquila chrysaetos* ) (Dang et al. 2025; Kang et al. 2007).

### 2.2 Data Sources and Processing Methods

Occurrence data for the forest musk deer were derived from two systematic field surveys: (1) an initial survey conducted between 2016 and 2017 across the three nature reserves and their surrounding areas, deploying a total of 120 transects; and (2) a subsequent survey conducted from May to September 2024 in the study area, deploying an additional 40 transects. To facilitate representative sampling, transects were randomly allocated within potential occurrence areas delineated from historical reserve records. Given the rugged terrain of the study area, our survey protocol required field teams to navigate across varied topographic gradients rather than tracking along highly accessible pathways. This effort typically incorporated diagonal or transverse searches across steep mid‐slopes, where terrain permitted. Each transect maintained a minimum length of 2 km, a 5 m search width on either side, and an inter‐transect spacing of approximately 2 km. Given the sympatric distribution of Siberian roe deer and the morphological similarity between their feces and those of the forest musk deer, we applied strict DNA molecular identification to eliminate observational errors (Tang et al. 2018). Fresh fecal samples were collected using sterile gloves, desiccated with silica gel, transported via cold chain, and stored at −20°C. Following laboratory DNA extraction and sequencing, only samples genetically confirmed as forest musk deer were retained. Each geographic coordinate of a verified fecal sample was recorded as an independent occurrence, representing the confirmed presence of the species rather than a distinct individual. This protocol ultimately yielded a total of 265 initial occurrence points.

To reduce model overfitting caused by spatial autocorrelation (Dormann et al. 2007), we performed spatial filtering based on the minimum recorded forest musk deer home range (~5 ha) (Table 1) (Green 1986; Gao 1986; Liu and Sheng 2000; Sheng and Liu 2007; Liu et al. 2020): we retained one random occurrence point per 120 m × 120 m grid cell using the SDM Toolbox in ArcGIS, resulting in 177 filtered points for modeling (Zheng et al. 2026).

**TABLE 1:** Criteria for selecting the scale.

Radius of scale	Home range area	Source
120 m	5.5 ha	The year‐round home range of the forest musk deer on the islands of Zhoushan City, Zhejiang Province (Liu and Sheng 2000)
180 m	10 ha	The year‐round home range of the western forest musk deer in Sichuan Province (Gao 1986)
360 m	40 ha	Primary home range of the forest musk deer in Shaanxi Province throughout the year (Liu et al. 2020)
720 m	168 ha	The year‐round home range of the forest musk deer in Shaanxi Province (Liu et al. 2020)
960 m	300 ha	The year‐round home range of the Himalaya musk deer in India (Green 1986)
1920 m	1256.64 ha	Maximum home range scale radius (Green 1986)

Based on previous studies regarding the habitat preferences and ecological requirements of forest musk deer (Gao et al. 2023; Jiang et al. 2020; Li et al. 2025; Liu et al. 2024; Sheng and Liu 2007), a total of 15 environmental variables across five distinct categories—climate, topography, vegetation, human disturbance, and water resources—were initially selected as potential predictors. Detailed information regarding the data sources, spatial resolutions, and specific preprocessing procedures (e.g., trigonometric transformation of aspect) is provided in the Supporting Information (Table S1).

All variables were preprocessed in ArcGIS 10.8: we performed multi‐scale optimization for non‐distance variables across spatial scales corresponding to musk deer home range sizes (5.5 ha to 1256.64 ha) (Table 1). Specifically, we conducted neighborhood processing for all environmental variable rasters across the specified scales using the Focal Statistics tool in ArcGIS 10.8 (ESRI, Redlands, CA, USA). For the categorical variable (Landcover), we calculated the majority class within each neighborhood, while for all other continuous variables, we calculated the mean value within each neighborhood (Bellamy and Altringham 2015). Finally, all environmental variable rasters were reprojected to the Universal Transverse Mercator (UTM) projection (Zone 49 N), clipped to the uniform spatial extent of the study area, and standardized to a 30 m grid cell size.

Ethics approval was not required for this study as it relied solely on non‐invasive data collection methods (e.g., field surveys of fecal occurrence points) and historical reserve records. No live animals were captured, handled, or disturbed during the course of this research.

### 2.3 Habitat Suitability Model

When modeling habitat suitability for cryptic species, addressing imperfect detection and potential false absences is a critical methodological consideration (Lahoz‐Monfort et al. 2014; Guillera‐Arroita 2017). Although spatial occupancy models effectively account for imperfect detection, they strictly require temporally replicated detection‐nondetection data at each sampling site within a closed population period (MacKenzie et al. 2002). Given our broad‐scale spatial survey design and the core objective of generating a continuous, high‐resolution habitat suitability surface for landscape connectivity modeling, we employed an ensemble species distribution modeling (SDM) framework. To mitigate biases associated with false absences without an explicit occupancy framework, we utilized a presence‐background modeling design. Because definitive true absences are practically unattainable for cryptic species and are highly susceptible to imperfect detection, we generated diverse configurations of pseudo‐absences to robustly capture background environmental availability (detailed below). Additionally, to ensure high data quality, false positives and spatial autocorrelation were strictly controlled via DNA molecular identification and spatial filtering, respectively (Section 2.2). The complete analytical workflow—encompassing data preparation, multi‐scale variable optimization, ensemble modeling, and subsequent landscape connectivity analysis—is visually summarized in Appendix Figure S1.

We first built univariate models for each variable at all tested scales in R v.4.4.2 using the “biomod2” package (v.4.2–6) (Thuiller et al. 2009), selecting the optimal scale for each variable based on the Area Under the receiver operating characteristic Curve (AUC) and True Skill Statistic (TSS) performance metrics (Allouche et al. 2006). To mitigate multicollinearity among variables at their optimal scales, we initially examined pairwise Pearson correlations (r) to identify potentially collinear variable pairs. Subsequently, we calculated Variance Inflation Factors (VIF), adopting a threshold of VIF < 5 for final variable retention (Dormann et al. 2013). When excluding collinear predictors, we prioritized variables based on their direct ecological relevance to the forest musk deer and their representation of distinct environmental constraints. We relied on higher AUC values from univariate models only when the ecological relevance between candidate variables was comparable (Bellamy and Altringham 2015).

Ensemble SDM was constructed by integrating 11 individual model algorithms (full list in Table S2) using “biomod2”. The final ensemble model was assembled from the highest‐performing candidates among these 11 algorithms (Thuiller et al. 2009). Compared to single‐algorithm approaches, ensemble modeling effectively mitigates prediction biases and spatial uncertainties, offering a more robust framework for delineating critical habitats and informing conservation planning for endangered species (Hao et al. 2019; Valavi et al. 2021). Given the absence of reliable confirmed absence records for wild forest musk deer, we generated pseudo‐absences using a random background selection approach. Specifically, to account for the uncertainty inherent in spatial sampling and to robustly capture environmental availability across varying presence‐to‐background ratios, we generated eight pseudo‐absence sets (two replicates each of 300, 500, 800, and 1000 points). These points were randomly distributed across the study area, explicitly excluding grid cells with recorded occurrences. For each model run, we randomly partitioned 80% of the forest musk deer occurrence points and all pseudo‐absence points into a training dataset for model fitting, reserving the remaining 20% for performance evaluation. Each algorithm was replicated 20 times across independent data partitions and all pseudo‐absence configurations. This rigorous cross‐validation strategy enabled the final ensemble to effectively buffer the stochasticity associated with individual background samples. We only retained individual models with mean AUC > 0.9 and mean TSS > 0.75 for ensemble construction (Eskildsen et al. 2013), using the standard AUC‐weighted mean (EMwmean) algorithm built into biomod2 as the ensemble rule. We then calculated mean variable contribution rates and response curves for predictors, and generated a 0–1 continuous habitat suitability map for the study area.

Based on the outputs of the final ensemble SDM, we identified primary habitats of forest musk deer via a two‐step procedure: first, we converted the continuous suitability output to a binary suitable/unsuitable map using the Lowest Presence Threshold (LPT, specifically the LPT90% threshold), which excludes the 10% of occurrence points with the lowest predicted suitability to balance omission error and overprediction (Pearson et al. 2007; Waltari and Guralnick 2009). Second, we retained only suitable patches ≥ 76 ha as primary habitat patches, corresponding to the average forest musk deer home range (Liu et al. 2020). We then calculated the overlap between these primary habitats and existing protected area boundaries to assess conservation gaps.

### 2.4 Landscape Connectivity Analysis

We first constructed a dispersal resistance surface from the ensemble SDM suitability output using a widely validated negative exponential function (Keeley et al. 2016):

(1) $$R=100-99\times \left[1-\exp\left(-c\times h\right)\right]/\left[1-\exp\left(-c\right)\right]$$

where *R* denotes the dispersal resistance value, and *h* denotes the habitat suitability value derived from the ensemble SDM. The parameter *c* determines the degree of non‐linearity, specifically governing the steepness of the curve. Ecologically, the forest musk deer is a typical habitat specialist with stringent environmental requirements for establishing primary home ranges (Liu et al. 2020; Sheng and Liu 2007). However, empirical studies demonstrate that such specialists typically exhibit increased tolerance for suboptimal matrices during long‐distance dispersal or prospecting movements (Cao et al. 2020; Keeley et al. 2016). Consequently, dispersal resistance remains relatively low across intermediate habitats, increasing abruptly only in critically unsuitable environments. This highly non‐linear relationship is well‐supported by telemetry and genetic data, with optimal *c* values ranging from 2 to 32 for various sensitive taxa (Keeley et al. 2017; Trainor et al. 2013). Consistent with these broad ecological patterns, we parameterized our baseline connectivity model using *c* = 8. Furthermore, to rigorously evaluate the robustness of our spatial predictions against parameter uncertainty, we conducted a sensitivity analysis across a broader parameter gradient, testing *c* values of 4, 6, 8, 10, 12, and 16.

Subsequently, we built potential dispersal corridors based on Circuit Theory using the Linkage Mapper v.2.0 toolbox (McRae and Kavanagh 2011), and evaluated corridor quality using the ratio of cost‐weighted distance to Euclidean distance (CWD: EucD). To further prioritize conservation and restoration efforts, we identified connectivity pinch points (critical dispersal bottlenecks) using the Pinchpoint Mapper module and Circuitscape v4.0.5 (McRae et al. 2013). We applied a cost‐weighted distance (CWD) cutoff threshold of 10,000 m to define the effective width of the corridors for the pinch point analysis. This threshold was parameterized to explicitly reflect the spatial ecology of the forest musk deer. Given an average home range diameter of approximately 1000 m (derived from our 76 ha primary patch threshold), and assuming a moderate resistance value of 10 in suboptimal habitats, a CWD of 10,000 conceptually permits a lateral detour of up to 1000 m when dispersing individuals encounter localized barriers. This parameterization accommodates plausible exploratory movements around obstacles while restricting excessive current diffusion into highly unsuitable matrices. Furthermore, an “all‐to‐one” simulation mode was utilized to calculate cumulative current density across the corridor network. Regions exhibiting disproportionately high current densities were defined as key pinch points. These areas function as critical hubs in the landscape where degradation could severely compromise the overall connectivity of the study area (McRae et al. 2008).

## 3 Results

### 3.1 Variable Pre‐Screening

Following multi‐scale optimization, our initial evaluation of the 15 candidate variables at their optimal spatial scales revealed substantial multicollinearity among several macro‐environmental predictors (Figure S2; Table S4). To ensure model robustness, we systematically excluded highly collinear variables (e.g., Bio1, Bio2, and elevation). During this screening procedure, variable retention was prioritized based on stronger ecological relevance to forest musk deer and higher univariate predictive performance (AUC) (Table S3).

Notably, the maximum temperature of the warmest month (Bio5) and annual precipitation (Bio12) exhibited a moderate pairwise correlation (|*r*| = 0.77; Figure S2). Both variables were retained because they represent distinct biophysical processes critical to this peripheral population of forest musk deer: Bio5 imposes direct physiological limits related to summer heat tolerance, whereas Bio12 acts as a macro‐environmental driver shaping essential forest habitats. To mitigate overall multicollinearity, we excluded the precipitation of the driest month (Bio14), a variable exerting less direct physiological limitation on forest musk deer compared to summer heat stress. Following this exclusion, VIF values for all remaining predictors dropped strictly below the threshold of 5 (Table S4). Ultimately, eight variables were retained for the final ensemble SDM construction: aspect, maximum temperature of the warmest month (Bio5), annual precipitation (Bio12), landcover, slope, distance to nearest road (Dis_road), distance to nearest river (Dis_river), and distance to nearest residential area (Dis_resident).

### 3.2 Environmental Variable Importance and Response Patterns

The relative importance of variables derived from the ensemble SDM showed that slope, Bio12, and Bio5 were the dominant drivers of forest musk deer habitat suitability, with relative contributions of 56.2%, 19.1%, and 12.8%, respectively, while the individual contributions of all remaining variables were each less than 5%. Full relative contribution values for all retained variables are provided in Table S5.

**FIGURE 2:**
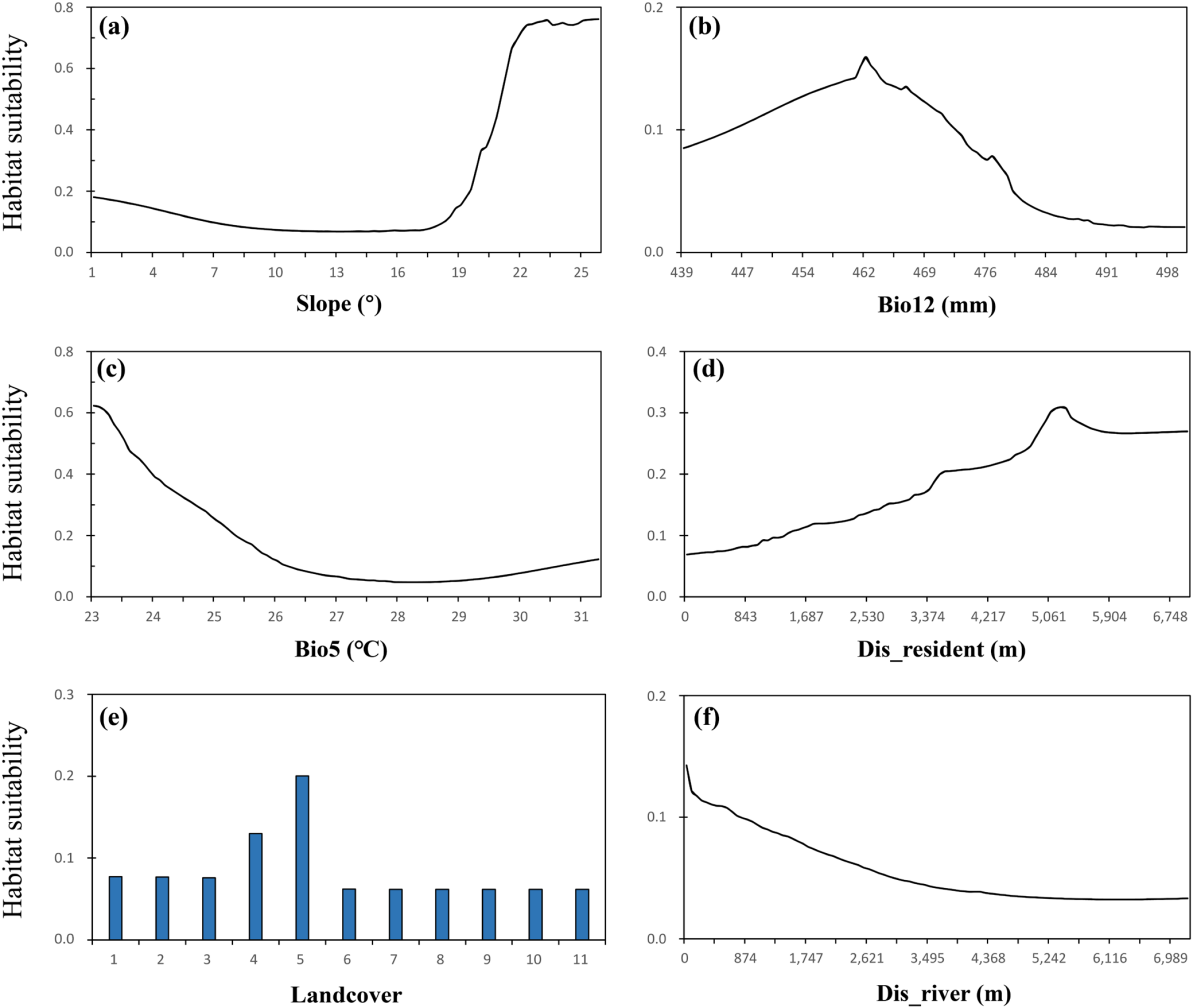
Response curves of habitat suitability for most influential variables. Response curves of habitat suitability to most influential environmental variables based on the multi‐scale ensemble model: (a) Slope, (b) Bio12, (c) Bio5, (d) Dis_resident, (e) Landcover, (f) Dis_river. For the landcover variable (e), values ranging from 1 to 11 correspond to: 1 = rainfed cropland, 2 = herbaceous cover cropland, 3 = irrigated cropland, 4 = closed deciduous broadleaved forest, 5 = closed evergreen needle‐leaved forest, 6 = grassland, 7 = marsh, 8 = flooded flat, 9 = impervious surfaces, 10 = bare areas, and 11 = water body.

The response curves for the six most influential predictors (Figure 2) revealed distinct habitat response patterns for forest musk deer: the species preferred steep slopes > 22°, areas > 5000 m away from residential settlements, and areas in close proximity to rivers. Furthermore, occurrence probability peaked at an annual precipitation of ~462 mm, and increased gradually as the maximum warm‐month temperature dropped below 27°C Regarding vegetation, forest musk deer showed the highest preference for coniferous forests (Landcover class 5), followed by broad‐leaved forests (Landcover class 4). Response curves for the remaining minor variables (Aspect and Dis_road) are provided in Figure S3.

### 3.3 Habitat Suitability and Primary Habitat Patches

Based on initial evaluations, we selected six high‐performance algorithms (RF, GBM, MARS, MaxEnt, GLM, and CTA) to construct the final ensemble SDM. These selected models exhibited high cross‐validated accuracy, with mean ROC values ranging from 0.908 (CTA) to 0.999 (RF), and mean TSS values from 0.757 (GLM) to 0.988 (RF) (Table S6). Importantly, our multi‐configuration testing revealed that model performance remained highly stable across the eight pseudo‐absence sets (mean ROC: 0.929–0.947; mean TSS: 0.781–0.804; Table S7). By integrating these models via a weighted average, the final ensemble effectively buffers individual algorithmic variance, providing a robust prediction for continuous habitat suitability mapping.

**FIGURE 3:**
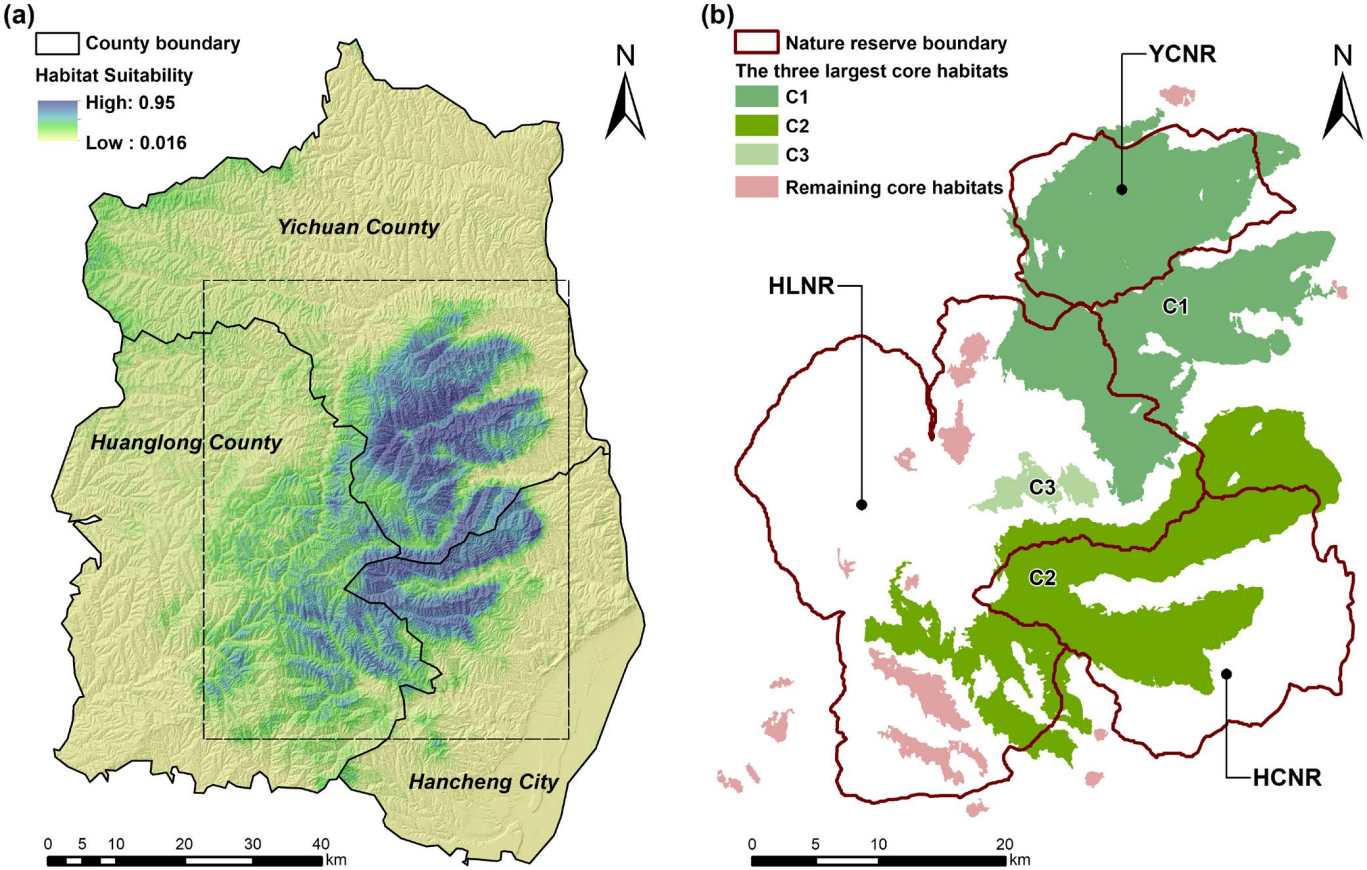
Spatial distribution of habitat suitability and primary habitats for the forest musk deer in the Huanglong Mountains. (a) Continuous habitat suitability map derived from the ensemble model. (b) Extraction of the primary habitats and their spatial overlap with existing nature reserves. The dashed box in (a) corresponds to the spatial extent of (b). C1, C2, and C3 represent the three largest primary habitat patches. HLNR: Shaanxi Yan'an Huanglong Mountains Brown Eared Pheasant National Nature Reserve; HCNR: Shaanxi Hancheng Huanglong Mountains Brown Eared Pheasant National Nature Reserve; YCNR: Shaanxi Yichuan Siberian Musk Deer Provincial Nature Reserve.

The ensemble SDM revealed that continuous suitable habitat for the forest musk deer is widely distributed across the Huanglong Mountains, with the highest‐suitability patches concentrated in Yichuan County (Figure 3a). In total, we identified 19 primary habitat patches encompassing an area of 863.0 km^2^ (Figure 3b; Table S8). Overall, 66.1% (570.1 km^2^) of these primary habitat areas overlap with existing protected areas, leaving 33.9% outside formal protection boundaries. The three largest patches (C1, C2, and C3) cover 421.4, 362.5, and 21.1 km^2^, respectively. While C3 is entirely encompassed by the current reserve network, formal protection covers only 62% of C1 and 79% of C2. Consequently, the unprotected sections of C1 and C2 represent major spatial conservation gaps. Specifically, C1 contains the largest unprotected area and should be the top priority for protected area expansion.

### 3.4 Potential Dispersal Corridors

**FIGURE 4:**
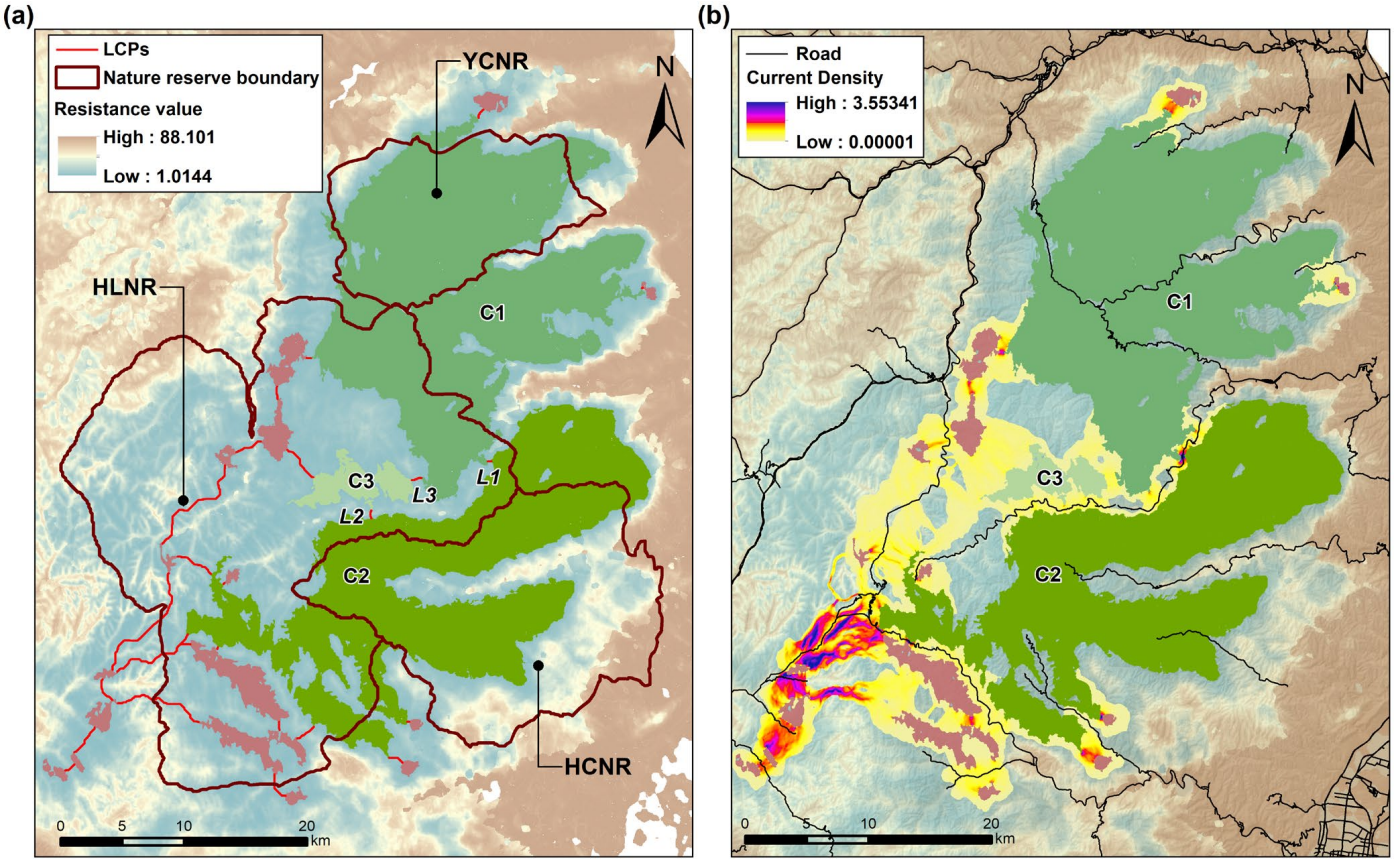
Habitat connectivity and potential conservation corridors for the forest musk deer. (a) Habitat resistance surface and the spatial distribution of least‐cost pathways (LCPs). L1, L2, and L3 represent the three most critical potential corridors connecting the three largest primary habitats. (b) Current density map identifying pinch points (bottlenecks) within the potential corridors where conservation efforts should be prioritized. C1, C2, and C3 represent the three largest primary habitat patches. HLNR: Shaanxi Yan'an Huanglong Mountains Brown Eared Pheasant National Nature Reserve; HCNR: Shaanxi Hancheng Huanglong Mountains Brown Eared Pheasant National Nature Reserve; YCNR: Shaanxi Yichuan Siberian Musk Deer Provincial Nature Reserve.

We identified 25 potential dispersal corridors connecting primary habitat patches based on Circuit Theory (Figure 4a). The least‐cost path (LCP) lengths of these corridors ranged from 72 to 10,804 m (mean: 2774.7 m), while the Euclidean distance between connected primary patches ranged from 30 to 8991 m (mean: 2332.8 m). Among these, three key corridors (L1, L2, and L3) connect the three largest primary patches. Under the baseline resistance scenario (*c* = 8), their cost‐weighted distance to Euclidean distance (CWD: EucD) ratios increased sequentially: L1 (3.74), L3 (4.73), and L2 (7.07). Given that a higher CWD: EucD ratio generally reflects greater landscape resistance and detour costs relative to an ideal straight‐line path, this sequence indicates a relative reduction in connectivity quality across these pathways.

Current density simulations (Figure 4b) identified multiple pinch points (critical dispersal bottlenecks) across the corridor network. Sensitivity analysis across a parameter gradient (*c* = 4 to 16) demonstrated that the overall spatial configuration of these primary corridors and key pinch points remained consistent (Figure S4). While an increase in the parameter *c* broadened the current density surface and laterally expanded pinch point areas, the geographic core locations of these bottlenecks did not shift. Among the three primary corridors (L1, L2, and L3), prominent pinch points consistently emerged along L1, which exhibited the highest current density. Notably, these critical bottlenecks along L1 are directly transected by an existing road. In contrast, current flow along L2 and L3 remained relatively dispersed. Additionally, a dense concentration of bottlenecks was observed in the pathways connecting patch C2 with the southwestern patches. These southwestern pinch points predominantly flank a northeast‐southwest expressway, with occasional spatial overlap (Figure 4b).

## 4 Discussion

### 4.1 Multi‐Scale Environmental Drivers and Spatial Adaptation Patterns

Understanding how endangered habitat specialists persist and adapt at their environmental margins represents a fundamental challenge in contemporary conservation biology. According to the center‐periphery hypothesis, populations at the extreme edges of a species' geographic distribution typically encounter sub‐optimal environmental conditions, rendering them highly susceptible to demographic stochasticity and localized extirpations (Eckert et al. 2008; Pironon et al. 2017). However, these edge populations also harbor unique adaptive potential and can serve as critical climate refugia under scenarios of rapid global environmental change (Lesica and Allendorf 1995; Rehm et al. 2015). By integrating multi‐scale ensemble species distribution models with circuit theory, our study elucidates the specific spatial ecological patterns of the forest musk deer at its northernmost known limit. Our results reveal that the persistence of this peripheral population is heavily contingent upon specific topographic and thermal refugia. Furthermore, we identify a critical spatial conflict at the landscape level: while steep, rugged terrains naturally buffer these key refugia from broad‐scale anthropogenic disturbances, the vital dispersal corridors connecting them are compromised by localized linear infrastructure. This fragmentation exposes significant conservation gaps within the current protected area network (Tucker et al. 2018; Zeller, Wattles, et al. 2020).

The close association of the forest musk deer with specific topographic and thermal refugia appears to be fundamentally shaped by the species' inherent ecophysiological constraints. As demonstrated by our ensemble SDM, steep terrains, adequate precipitation regimes, and summer thermal limits act as the dominant environmental drivers defining habitat suitability at this northern margin. These environmental associations reflect the forest musk deer's specialized adaptations (Sheng and Liu 2007). Specifically, the thermal intolerance of forest musk deer, driven by highly insulating pelage, necessitates the selection of cool refugia. Simultaneously, their distinctive locomotor agility allows them to exploit steep terrains as secure strongholds to evade sympatric predators, such as leopards ( *Panthera pardus* ) and yellow‐throated martens ( *Martes flavigula* ). To satisfy these dual requirements of security and thermal regulation, adequate annual precipitation plays a critical mediating role by facilitating the development of closed‐canopy coniferous and mixed broad‐leaved forests in this otherwise semi‐arid region. These dense forest stands not only supply essential primary food sources, such as tender foliage, lichens, and mosses, but also create a crucial microclimatic buffering effect that mitigates summer thermal extremes (De Frenne et al. 2019).

Our multi‐scale optimization framework reveals a scale‐dependent divergence that contrasts with traditional species‐environment assumptions. In classical ecological theory, broad‐scale hydro‐thermal factors typically constrain species distribution at the macro‐level, while topography defines local‐scale habitat suitability (Mackey and Lindenmayer 2001; Pearson and Dawson 2003). However, our scale optimization revealed a contrasting spatial pattern for this peripheral population. The association with steep slopes operates optimally at a broad spatial scale (1920 m radius), essentially encompassing the entire home range of forest musk deer (Table 1). This suggests that steep terrain serves as a fundamental landscape‐level prerequisite, upon which forest musk deer likely rely to ensure broad‐scale security from anthropogenic disturbances and predators. Conversely, the response to the maximum temperature of the warmest month operates at a localized, fine‐grained scale (120 m radius). This suggests that while the regional macro‐climate permits widespread occurrence, the physiological heat intolerance of forest musk deer likely necessitates specific micro‐habitat conditions. To avoid physiological heat stress from high summer temperatures exceeding their thermal tolerance, forest musk deer preferentially utilize habitats with shaded aspects and dense coniferous canopy cover (Sheng and Liu 2007). Together, these scale‐specific responses highlight a decoupled spatial adaptation pattern: balancing broad‐scale security with micro‐scale thermal regulation at this northern environmental margin. However, we caution that such scale‐environment relationships often exhibit spatial non‐stationarity (Osborne et al. 2007). The optimal scales identified herein are likely context‐dependent (Boyce 2006; Mysterud and Ims 1998), reflecting the unique biophysical constraints of this northern landscape. Whether forest musk deer residing in other distribution ranges (e.g., southwestern China) exhibit parallel multi‐scale dependencies warrants further comparative investigations.

Our ensemble SDM indicates that anthropogenic disturbance variables contributed minimally to overall habitat suitability. This finding contrasts with studies from highly fragmented, human‐dominated landscapes, in which human disturbance factors frequently emerge as the primary limiting factors for wild ungulate occurrence (Benítez‐López et al. 2010; Nellemann et al. 2003; Rowland et al. 2000). However, this low statistical contribution should not be misinterpreted as species' insensitivity to human activities. Instead, it likely reflects a landscape‐scale spatial segregation driven by the region's steep topography. Geomorphologically, the Huanglong Mountains are characterized by deep gullies and a complex “valley‐slope‐ridge” structure (Li et al. 2012). This terrain likely acts as a physical barrier that largely confines agricultural expansion and residential activities to the lower, accessible valleys, leaving the steep mid‐to‐upper slopes relatively intact. Bolstered by regional forest conservation policies and the cessation of commercial logging, this topographic isolation has facilitated the preservation of extensive, high‐quality habitats. Ultimately, this spatial segregation of human activity and wildlife habitat likely underpins the area's capacity to support this population, allowing forest musk deer to persist despite residing at their northern distribution margin (Nellemann et al. 2003).

### 4.2 Conservation Gaps and Spatial Conflicts at Dispersal Bottlenecks

While topographic isolation buffers these refugia, their spatial fragmentation exposes an underlying vulnerability in regional landscape connectivity. Our spatial overlay indicates that approximately 34% of high‐quality primary habitats, including substantial portions of the two largest patches (C1 and C2), remain outside current statutory boundaries. Historically, these protected areas were established primarily for the conservation of avian flagship species, such as the Brown Eared Pheasant ( *Crossoptilon mantchuricum* ), whose ecological niche and habitat requirements differ substantially from those of forest‐dwelling ungulates (Li et al. 2020).

Furthermore, current density analysis identified critical dispersal bottlenecks (pinch points) along the primary inter‐patch corridors, particularly within the L1 corridor, which links the largest primary patches. In mountainous regions, human linear infrastructure (e.g., highways) is frequently constructed along relatively gentle valley bottoms. Therefore, forest musk deer are forced to traverse these disturbed zones during dispersal, resulting in a significant spatial conflict (Gibeau et al. 2002; Tucker et al. 2018).

This localized conflict exemplifies a pervasive global trend characterizing the Anthropocene: expanding transportation infrastructure increasingly disrupts the historical dispersal routes of terrestrial mammals (Ibisch et al. 2016; Tucker et al. 2018). For sensitive taxa such as the forest musk deer, roads are more than mere physical barriers causing direct mortality (roadkill). These linear infrastructures also generate extensive “zones of influence” driven by chronic noise, artificial lighting, and pollution, thereby triggering strong behavioral avoidance (Fahrig and Rytwinski 2009; Forman and Alexander 1998). Over time, the functional severance of these valley corridors has the potential to restrict individual dispersal, disrupt seasonal foraging movements, and impede inter‐patch gene flow (Kuehn et al. 2006). Left unmitigated, this isolation potentially escalates the risks of inbreeding depression and renders these patch‐dwelling groups vulnerable to stochastic environmental events or localized disease outbreaks (Balkenhol and Waits 2009; Holderegger and Di Giulio 2010). Ultimately, the spatial intersection of high‐current‐density ecological bottlenecks with the existing road network confirms that linear infrastructure represents the primary barrier hindering landscape permeability for forest musk deer.

### 4.3 Implications for Landscape Conservation Planning

Translating spatial ecological findings into actionable policy is critical for the systematic preservation of peripheral populations (Arlettaz et al. 2010; Cook et al. 2013). Securing the long‐term adaptive potential of the forest musk deer in the Huanglong Mountains calls for a transition from static, isolated patch‐based protection to dynamic, network‐oriented landscape management (Kukkala and Moilanen 2013; Margules and Pressey 2000). To achieve this, we propose the following targeted recommendations:

1. Strategic expansion of protected areas. We recommend expanding the existing provincial and national nature reserve boundaries to formally encompass the remaining 34% of unprotected primary habitats identified in this study. High priority should be placed on preserving the steep, coniferous‐dominated refugia (Keppel et al. 2012; Morelli et al. 2016).
2. Mitigation of linear infrastructure barriers. Mitigating the identified dispersal bottlenecks represents a key management objective. Targeted, ecologically sound crossing structures, such as well‐vegetated wildlife underpasses or overpasses tailored to the behavioral traits of forest musk deer, should be integrated into the existing road networks where they intersect primary corridors (Clevenger and Waltho 2005; Corlatti et al. 2009). Special priority must be given to corridor L1 and the southwestern bottleneck cluster flanking the elevated expressway. This should be supported by regulations restricting further infrastructure expansion, mining, or intensive tourism development within these vulnerable valley bottlenecks.
3. Cross‐administrative collaborative governance. Establishing a collaborative management framework among the three distinct nature reserves is essential to unify anti‐poaching patrols, standardize habitat monitoring protocols, and facilitate holistic landscape planning. This integration will effectively mitigate the spatial scale mismatches inherent in fragmented governance (Bodin 2017; Dallimer and Strange 2015).
4. Community engagement and ecological compensation. The long‐term success of these spatial conservation measures relies heavily on the support of local communities (Bodin 2017). Integrating community‐based resource monitoring with ranger‐led patrols can foster local ownership and lead to more effective conservation enforcement (Danielsen et al. 2009). Additionally, establishing comprehensive ecological compensation mechanisms that account for both direct and indirect costs (such as wildlife‐induced damage) can help alleviate potential economic losses resulting from land‐use restrictions, thereby mitigating the local livelihood burdens of human‐wildlife conflicts and encouraging active participation in conservation efforts (Hou et al. 2021; Liu and Dou 2022; Yang et al. 2020).

### 4.4 Limitations and Future Perspectives

While this study provides a multi‐scale analytical framework for evaluating habitat and connectivity at range margins, several limitations should be noted.

First, conceptualized within Hutchinson's n‐dimensional hypervolume framework (Hutchinson 1957), our ensemble SDM primarily captures the fundamental niche of the forest musk deer based on Grinnellian abiotic predictors (Grinnell 1917). These models do not explicitly account for Eltonian dimensions (Elton 1927), such as complex biotic interactions (e.g., potential interspecific competition with sympatric Siberian roe deer or predation pressure from apex predators) (Araújo and Luoto 2010; Soberón 2010; Wisz et al. 2013).

Second, regarding background data, our reliance on randomly selected pseudo‐absences represents a potential methodological limitation. Although we implemented spatial filtering to mitigate sampling autocorrelation, standard random background selection does not inherently account for all potential spatial biases as effectively as target‐group background selection might. Consequently, this approach may still introduce minor spatial biases into the final habitat assessments. Additionally, random background selection may misclassify unsurveyed but suitable habitats as unsuitable, reflecting the broader challenge of imperfect detection and false absences in wildlife surveys (Guillera‐Arroita 2017; Lahoz‐Monfort et al. 2014).

Third, while circuit theory offers spatially explicit predictions of movement probabilities based on landscape resistance, the model provides only a static representation of landscape connectivity. Future studies should incorporate dynamic temporal variables, such as varying snow cover or fluctuating human traffic volumes, to map how corridor permeability shifts seasonally (Bishop‐Taylor et al. 2018; Zeigler and Fagan 2014; Zeller, Lewison, et al. 2020).

Finally, the simulated dispersal corridors and pinch points identified in this study remain theoretical constructs requiring empirical validation. Future empirical research integrating non‐invasive landscape genetics via extensive fecal DNA sampling and GPS telemetry will be critical to quantify actual rates of gene flow and document the physical movement trajectories of individual forest musk deer across these anthropogenic barriers (Cushman et al. 2006; Sawaya et al. 2013; Zeller et al. 2012). Addressing these knowledge gaps and prioritizing both the preservation of thermal refugia and the mitigation of infrastructural barriers will better equip conservation practitioners to sustain the long‐term viability of this endangered specialist at its northern range margins.

## 5 Conclusions

In conclusion, this multi‐scale ensemble approach elucidates the spatial adaptation patterns of the endangered forest musk deer at its northernmost known distribution margin. We highlight three key conservation insights:

1. Forest musk deer exhibit a decoupled spatial pattern, relying on extensive steep terrain at the macro‐scale for security while utilizing specific micro‐scale thermal conditions (i.e., localized summer temperature limits) as microclimatic refugia to mitigate physiological heat stress.
2. Substantial conservation gaps exist, with approximately 34% of the identified primary habitats currently lacking statutory protection. Furthermore, critical inter‐patch dispersal bottlenecks spatially intersect with or closely flank existing linear infrastructure along valley bottoms.
3. To secure the long‐term viability of this peripheral population, conservation planning should transition from static, isolated protection toward dynamic, network‐oriented landscape management. Expanding existing protected areas to formally encompass these unprotected habitats represents a fundamental first step. Furthermore, mitigating linear infrastructure barriers requires targeted crossing structures, particularly along corridor L1 and the southwestern bottleneck cluster. Finally, establishing cross‐administrative collaborative governance and fostering local community engagement are essential to sustain this endangered specialist at its northern environmental margin.

## Author Contributions

**Wangshan Zheng:** conceptualization (equal), data curation (equal), formal analysis (lead), investigation (equal), methodology (equal), visualization (equal), writing – original draft (lead), writing – review and editing (equal). **Pengfei Luo:** conceptualization (equal), data curation (equal), formal analysis (equal), investigation (equal), methodology (equal), writing – review and editing (equal). **Yixin Li:** data curation (equal), formal analysis (equal), investigation (equal), methodology (equal), visualization (equal), writing – review and editing (equal). **Luyao Hai:** data curation (equal), investigation (equal), methodology (equal), writing – review and editing (equal). **Xuelin Jin:** conceptualization (equal), project administration (equal), resources (equal), writing – review and editing (equal). **Xinghu Qin:** conceptualization (equal), funding acquisition (equal), project administration (equal), resources (equal), supervision (equal), writing – review and editing (equal). **Liping Yan:** funding acquisition (equal), project administration (equal), resources (equal), supervision (equal), writing – review and editing (equal). **Defu Hu:** conceptualization (equal), funding acquisition (lead), project administration (equal), resources (equal), supervision (lead), writing – original draft (equal), writing – review and editing (equal).

## Funding

This work was supported by the National Forestry and Grassland Administration (grant number 2024090701006) under the Species Specific Survey project.

## Conflicts of Interest

The authors declare no conflicts of interest.

## Supporting information


**Table S1:** Interpretation and sources of environmental variables.
**Table S2:** The model algorithms included in the “biomod2” (v 4.2‐6).
**Table S3:** The performance of the univariate SDM constructed at different scales.
**Table S4:** Variance Inflation Factor (VIF) values of candidate environmental variables before and after multicollinearity screening.
**Table S5:** Relative contribution rates (%) of the final retained environmental variables.
**Table S6:** Predictive performance of the nine modeling.
**Table S7:** Predictive performance of models across the eight pseudo‐absence (PA) sets.
**Table S8:** Spatial overlap and conservation coverage of the three largest primary habitat patches by existing nature reserves.
**Figure S1:** Schematic diagram of the complete multi‐scale ensemble SDM and landscape connectivity workflow. The framework illustrates five primary analytical phases: (1) data preparation, including non‐habitat masking; (2) multi‐scale optimization (via univariate biomod2 modeling) and rigorous variable screening to address multicollinearity (e.g., VIF assessment); (3) multi‐algorithm ensemble modeling utilizing the biomod2 R package across varied pseudo‐absence sets; (4) ensemble evaluation and weighting to generate continuous and binary habitat maps; and (5) landscape connectivity analysis, which integrates the derived primary habitats and a suitability‐inverted resistance surface (*c* = 8) to construct ecological corridors and identify critical dispersal pinch points.
**Figure S2:** Heatmap of Pearson correlation for all environmental variables at the optimal scale.
**Figure S3:** Response curves of habitat suitability for minor variables. Response curves of habitat suitability to the remaining minor environmental variables based on the multi‐scale ensemble model: (g) Aspect, (h) Dis_road.
**Figure S4:** Sensitivity analysis of landscape connectivity pinch points across a gradient of resistance parameter *c* values. Panels (a) through (f) depict the cumulative current density surfaces generated using c values of 4, 6, 8, 10, 12, and 16, respectively. Panel (c) represents the baseline parameterization (*c* = 8) used in the main analysis.

## Data Availability

The environmental variable layers, species occurrence data, and R scripts supporting the findings of this study are deposited in the Dryad Digital Repository (https://doi.org/10.5061/dryad.qbzkh1902; which will be activated upon publication). To protect this endangered and highly sensitive species from potential poaching risks, the precise spatial coordinates of the occurrence records have been appropriately coarsened prior to public release, in accordance with best practices for sensitive biodiversity data.
