# Supplementary material for: Assessing Habitat Suitability and Landscape Connectivity for the Endangered Forest Musk Deer (Moschus berezovskii) at Its Northern Distribution Margin: A Multi‐Scale Ensemble Approach
Source: Ecol Evol. 2026 Aug 12;16(8):e74086. doi: 10.1002/ece3.74086 (PMC13469347; doi:10.1002/ece3.74086)
Supplement: Supplementary file 1 [file ECE3-16-e74086-s001.docx]

**Appendix**

**Table S1**

Interpretation and sources of environmental variables.

| **Category** | **Variable** | **Interpretation** | **Data source** |
| --- | --- | --- | --- |
| Climate | Bio1 | Annual Mean Temperature (°C) | Sourced from the ERA5-Land dataset by the ECMWF. Downscaled and standardized by Prof. Liu Bintao's team at the Institute of Mountain Hazards and Environment, Chinese Academy of Sciences. |
|  | Bio2 | Mean Diurnal Range (Mean of monthly (max temp - min temp)) (℃) |  |
|  | Bio5 | Max Temperature of the Warmest Month (℃) |  |
|  | Bio6 | Min Temperature of the Coldest Month (℃) |  |
|  | Bio7 | Temperature Annual Range (℃) |  |
|  | Bio12 | Annual Precipitation (mm) |  |
|  | Bio14 | Precipitation of the Driest Month (mm) |  |
| Topography | Elevation | Elevation extracted from digital elevation map (m) | https://earthdata.nasa.gov/esds/srtm |
|  | Aspect | Calculated from DEM data | DEM |
|  | Slope | Calculated from DEM data | DEM |
| Vegetation | NDVI | Normalized Difference Vegetation Index. Quantifying vegetation cover and growth through remotely sensed data. | National Science & Technology Resource Sharing Service Platform |
|  | Landcover | Constructed using continuous change detection and spatiotemporal collaborative optimization techniques with full-time series Landsat imagery from Google Earth Engine, it features 30m spatial resolution and 35 fine land cover classes. | Derived from the first global long-term land cover dynamic dataset (1985-2022). |
| Human disturbance | Dis_road | Euclidean distance to nearest paved roads (m) | OpenStreetMap |
|  | Dis_resident | Euclidean distance to nearest residents (m) | OpenStreetMap |
| Water resources | Dis_river | Euclidean distance to nearest rivers (m) | OpenStreetMap |

**Note:** High-resolution temperature and precipitation data were obtained from the ERA5-Land dataset of the European Centre for Medium-Range Weather Forecasts (ECMWF), with a 30 m spatial resolution; these data represent the standard 1991-2020 climatological baseline means. Topographic data were derived from the Digital Elevation Model (DEM) acquired by the Shuttle Radar Topography Mission (SRTM) (https://earthdata.nasa.gov/esds/srtm), which has a 30 m spatial resolution and is referenced to the WGS84 geographic coordinate system. We used the surface analysis tools in the Spatial Analyst extension of ArcGIS 10.8 to calculate slope and raw aspect: slope is expressed in degrees (0°-90°) to represent the steepness of the terrain. To address statistical biases caused by the circular nature of raw aspect values (which range from 0° to 360°), we transformed aspect using the standard trigonometric method (Roberts & Cooper, 1989). Landcover was sourced from the GLC_FCS30D dataset, the world’s first long-time-series global land cover dynamic dataset developed by the Aerospace Information Research Institute, Chinese Academy of Sciences (AIRCAS). This dataset was generated using full-time-series open-access Landsat imagery (2022) on the Google Earth Engine platform, with a continuous change detection and spatiotemporal collaborative optimization framework, and has a 30 m spatial resolution (Zhang et al., 2024). In this study, the Landcover data were reclassified into 11 major categories using ArcGIS (namely: rainfed cropland, herbaceous cover cropland, irrigated cropland, closed deciduous broadleaved forest, closed evergreen needle-leaved forest, grassland, marsh, flooded flat, impervious surfaces, bare areas, water body). Normalized Difference Vegetation Index (NDVI) data were obtained from the National Science & Technology Resource Sharing Service Platform, generated by integrating full-time series Landsat 5/7/8/9 imagery on the Google Earth Engine platform via multi-source data fusion and time-series smoothing algorithms, with a 30 m spatial resolution (Yang et al., 2019). Rasters of distance to roads, water bodies, and residential areas were generated from vector layers of roads, water bodies, and building footprints extracted from the open-source OpenStreetMap (OSM) geospatial database. We calculated the straight-line Euclidean distance from each grid cell to the nearest major road, natural water body, and residential area using the Euclidean Distance tool in the Spatial Analyst extension of ArcGIS 10.8, with all output rasters resampled to a 30 m spatial resolution.

**Table S2**

The model algorithms included in the “biomod2” (v 4.2-6).

| **Prediction model category** | **Prediction model** |
| --- | --- |
| Regression-based models | Generalized linear model (GLM) |
|  | Generalized additive model (GAM) |
|  | Multivariate adaptive regression splines (MARS) |
|  | Flexible discriminant analysis (FDA) |
| Machine-learning models | Maximum entropy (MAXENT) |
|  | Generalized boosting model (GBM) |
|  | Random forest (RF) |
|  | Classification tree analysis (CTA) |
|  | Artificial neural network (ANN) |
|  | Extreme Gradient Boosting (XGBOOST) |
| Profile model | Surface range envelop (SRE) |

**Table S3**

The performance of the univariate SDM constructed at different scales

|  | **Aspect** | **Bio1** | **Bio2** | **Bio5** | **Bio6** | **Bio7** | **Bio12** | **Bio14** | **DEM** | **Land-cover** | **NDVI** | **Slope** |
| --- | --- | --- | --- | --- | --- | --- | --- | --- | --- | --- | --- | --- |
| **120m radius scale** | | | | | | | | | | | | |
| TSS | 0.696 | 0.623 | 0.578 | **0.690** | **0.673** | 0.650 | 0.580 | 0.409 | 0.589 | 0.372 | 0.590 | 0.548 |
| AUC | 0.895 | 0.883 | 0.875 | **0.914** | **0.901** | 0.906 | 0.884 | 0.769 | 0.872 | 0.696 | 0.883 | 0.839 |
| **180m radius scale** | | | | | | | | | | | | |
| TSS | 0.686 | **0.632** | 0.593 | 0.639 | 0.669 | 0.637 | 0.606 | 0.444 | **0.621** | 0.386 | 0.626 | 0.624 |
| AUC | 0.895 | **0.888** | 0.887 | 0.896 | 0.898 | 0.905 | 0.883 | 0.786 | **0.881** | 0.697 | 0.894 | 0.884 |
| **360m radius scale** | | | | | | | | | | | | |
| TSS | **0.757** | 0.561 | 0.617 | 0.618 | 0.587 | 0.654 | 0.597 | 0.461 | 0.582 | **0.387** | 0.634 | 0.636 |
| AUC | **0.932** | 0.866 | 0.884 | 0.892 | 0.877 | 0.911 | 0.889 | 0.801 | 0.859 | **0.704** | 0.897 | 0.902 |
| **720m radius scale** | | | | | | | | | | | | |
| TSS | 0.658 | 0.622 | 0.610 | 0.619 | 0.621 | **0.680** | **0.613** | 0.504 | 0.580 | 0.353 | **0.639** | 0.688 |
| AUC | 0.894 | 0.875 | 0.896 | 0.897 | 0.870 | **0.914** | **0.891** | 0.819 | 0.870 | 0.677 | **0.899** | 0.923 |
| **960m radius scale** | | | | | | | | | | | | |
| TSS | 0.634 | 0.595 | 0.663 | 0.631 | 0.663 | 0.675 | 0.615 | 0.551 | 0.616 | 0.371 | 0.636 | 0.692 |
| AUC | 0.863 | 0.873 | 0.904 | 0.900 | 0.890 | 0.913 | 0.888 | 0.847 | 0.878 | 0.687 | 0.890 | 0.933 |
| **1920m radius scale** | | | | | | | | | | | | |
| TSS | 0.591 | 0.554 | **0.660** | 0.630 | 0.638 | 0.648 | 0.610 | **0.650** | 0.558 | 0.383 | 0.614 | **0.698** |
| AUC | 0.845 | 0.869 | **0.912** | 0.909 | 0.897 | 0.907 | 0.887 | **0.880** | 0.874 | 0.696 | 0.888 | **0.938** |

**Note:** Taking both TSS and AUC into account, the univariate model with the highest AUC is considered the optimal scale for that variable and is shown in bold in the table.

**Table S4**

Variance Inflation Factor (VIF) values of candidate environmental variables before and after multicollinearity screening

| **Variables** | **Initial VIF (15 variables)** | **Final VIF (8 retained variables)** |
| --- | --- | --- |
| Aspect | 1.087 | 1.043 |
| Bio1 | 623.410 | - |
| Bio2 | 53.134 | - |
| Bio5 | 670.488 | 4.988 |
| Bio6 | 189.301 | - |
| Bio7 | 58.737 | - |
| Bio12 | 10.771 | 3.638 |
| Bio14 | 6.751 | - |
| Dis_resident | 2.176 | 1.851 |
| Dis_river | 1.275 | 1.133 |
| Dis_road | 1.465 | 1.366 |
| Elevation | 71.048 | - |
| Landcover | 1.579 | 1.229 |
| NDVI | 5.622 | - |
| Slope | 2.413 | 1.435 |

**Note:** Variables with a dash “-” were excluded to mitigate multicollinearity, with preference given to preserving variables with higher ecological relevance to the forest musk deer.

**Table S5**

Relative contribution rates (%) of the final retained environmental variables

| Variable | **Slope** | **Bio12** | **Bio5** | **Dis_resident** | **Landcover** | **Dis_river** | **Dis_road** | **Aspect** |
| --- | --- | --- | --- | --- | --- | --- | --- | --- |
| Contribution | **56.2** | **19.1** | **12.8** | 3.7 | 3.7 | 2.4 | 1.7 | 0.4 |

**Table S6**

Predictive performance of the nine modeling.

| **Algorithm** | **ROC (Mean ± SD)** | **TSS (Mean ± SD)** |
| --- | --- | --- |
| RF * | 0.999 ± 0.000 | 0.988 ± 0.007 |
| GBM * | 0.979 ± 0.005 | 0.868 ± 0.015 |
| MaxEnt * | 0.940 ± 0.018 | 0.786 ± 0.037 |
| MARS * | 0.939 ± 0.009 | 0.769 ± 0.024 |
| GLM * | 0.929 ± 0.008 | 0.757 ± 0.019 |
| CTA * | 0.908 ± 0.042 | 0.784 ± 0.067 |
| FDA | 0.928 ± 0.009 | 0.722 ± 0.023 |
| GAM | 0.920 ± 0.009 | 0.727 ± 0.019 |
| ANN | 0.907 ± 0.037 | 0.732 ± 0.065 |

**Note:** Values are presented as Mean ± Standard Deviation (SD). Algorithms marked with an asterisk (*) represent the six high-performance models selected to construct the final ensemble SDM (criteria: mean AUC > 0.9 and mean TSS > 0.75). ROC = Receiver Operating Characteristic curve; TSS = True Skill Statistic. Given the inclusion of the categorical environmental variable Landcover, the SRE and XGBoost models were automatically excluded during the operation of the “biomod2” package.

**Table S7**

Predictive performance of models across the eight pseudo-absence (PA) sets.

| **Pseudo-absence set** | **Number of points** | **ROC (Mean ± SD)** | **TSS (Mean ± SD)** |
| --- | --- | --- | --- |
| PA1 | 300 | 0.929 ± 0.044 | 0.786 ± 0.094 |
| PA2 | 300 | 0.936 ± 0.040 | 0.798 ± 0.084 |
| PA3 | 500 | 0.939 ± 0.036 | 0.790 ± 0.091 |
| PA4 | 500 | 0.946 ± 0.030 | 0.804 ± 0.082 |
| PA5 | 800 | 0.942 ± 0.035 | 0.798 ± 0.085 |
| PA6 | 800 | 0.937 ± 0.035 | 0.782 ± 0.094 |
| PA7 | 1000 | 0.947 ± 0.026 | 0.802 ± 0.085 |
| PA8 | 1000 | 0.936 ± 0.036 | 0.782 ± 0.091 |

**Note:** Values are presented as Mean ± Standard Deviation (SD). The eight pseudo-absence (PA) sets were generated using a gradient of 300, 500, 800, and 1000 points, with two replicates for each quantity. The narrow standard deviations and consistent evaluation metrics across different PA groups indicate that the model performance remained highly stable regardless of the varying background point quantities and their random spatial configurations.

**Table S8**

Spatial overlap and conservation coverage of the three largest primary habitat patches by existing nature reserves.

| **Primary habitat** | **Total Area** (km^2^) | **YCNR overlap** (km² / %) | **HLNR overlap** (km² / %) | **HCNR overlap** (km² / %) | **Unprotected area** (km² / %) |
| --- | --- | --- | --- | --- | --- |
| C1 | 421.4 | 173.0 (41.05%) | 89.0 (21.12%) | 0 (0%) | 159.4 (37.83%) |
| C2 | 362.5 | 0 (0%) | 107.3 (29.6%) | 179.7 (49.57%) | 75.5 (20.83%) |
| C3 | 21.1 | 0 (0%) | 21.1 (100%) | 0 (0%) | 0 (0%) |

**Note:**

HLNR: Shaanxi Yan'an Huanglong Mountains Brown Eared Pheasant National Nature Reserve;

HCNR: Shaanxi Hancheng Huanglong Mountains Brown Eared Pheasant National Nature Reserve;

YCNR: Shaanxi Yichuan Siberian Musk Deer Provincial Nature Reserve.

**Fig. S1. Schematic diagram of the complete multi-scale ensemble SDM and landscape connectivity workflow.**

The framework illustrates five primary analytical phases: (1) data preparation, including non-habitat masking; (2) multi-scale optimization (via univariate biomod2 modeling) and rigorous variable screening to address multicollinearity (e.g., VIF assessment); (3) multi-algorithm ensemble modeling utilizing the biomod2 R package across varied pseudo-absence sets; (4) ensemble evaluation and weighting to generate continuous and binary habitat maps; and (5) landscape connectivity analysis, which integrates the derived primary habitats and a suitability-inverted resistance surface (*c* = 8) to construct ecological corridors and identify critical dispersal pinch points.

**Fig. S2. Heatmap of Pearson correlation for all environmental variables at the optimal scale.**

**Fig. S3.** **Response curves of habitat suitability for minor variables.**

Response curves of habitat suitability to the remaining minor environmental variables based on the multi-scale ensemble model: (g) Aspect, (h) Dis_road.

**Fig. S4.** **Sensitivity analysis of landscape connectivity pinch points across a gradient of resistance parameter *c* values.**

Panels (a) through (f) depict the cumulative current density surfaces generated using c values of 4, 6, 8, 10, 12, and 16, respectively. Panel (c) represents the baseline parameterization (*c* = 8) used in the main analysis.

**References for the Appendix**

Roberts, D. W., & Cooper, S. V. (1989). Concepts and techniques of vegetation mapping. In D. E. Ferguson, P. Morgan, & F. D. Johnson (Comps.), *Proceedings—Land classifications based on vegetation: Applications for resource management* (General Technical Report INT-257, pp. 90-96). U.S. Department of Agriculture, Forest Service, Intermountain Research Station.

Yang, J., Dong, J., Xiao, X., Dai, J., Wu, C., Xia, J., Zhao, G., & Ge, Q. (2019). Divergent shifts in peak photosynthesis timing of temperate and alpine grasslands in China. *Remote Sensing of Environment*, *233*, 111395. https://doi.org/10.1016/j.rse.2019.111395.

Zhang, X., Zhao, T. T., Xu, H., Liu, W. D., Wang, J. Q., Chen, X. D., & Liu, L. Y. (2024). GLC_FCS30D: The first global 30 m land-cover dynamics monitoring product with a fine classification system for the period from 1985 to 2022 generated using dense-time-series Landsat imagery and the continuous change-detection method. *Earth System Science Data*, *16*(3), 1353-1381. <https://doi.org/10.5194/essd-16-1353-2024>.
